# The effects of antipsychotic medications on microbiome and weight gain in children and adolescents

**DOI:** 10.1186/s12916-019-1346-1

**Published:** 2019-06-19

**Authors:** Tali Bretler, Hagar Weisberg, Omry Koren, Hadar Neuman

**Affiliations:** 1 Ziv Medical Center, Derech Harambam St, 13100 Safed, Israel; 2 Azrieli Faculty of Medicine, Bar Ilan University, 8 Henrietta Szold St, 1311502 Safed, Israel; 3 Zefat Academic College, 11 Jerusalem St, 1320611 Safed, Israel

**Keywords:** Antipsychotic drugs, Second-generation antipsychotics, Microbiome, Adolescents, Weight gain

## Abstract

**Background:**

Atypical antipsychotics, also known as second-generation antipsychotics, are commonly prescribed as treatment for psychotic disorders in adults, as well as in children and adolescents with behavioral problems. However, in many cases, second-generation antipsychotics have unwanted side effects, such as weight gain, potentially further increasing risk for morbidities including obesity, diabetes, and cardiovascular disease. While various mechanisms for this weight gain have been proposed, including effects on metabolic hormone signaling, recent evidence points to the importance of the gut microbiome in this process. The microbial communities residing within the gut are affected by second-generation antipsychotics and can confer weight gain.

**Main text:**

This review summarizes recent findings and presents data linking second-generation antipsychotics, gut microbiota alterations and weight gain. The review focuses on children and adolescent populations, which have not previously received much attention, but are of great interest because they may be most vulnerable to gut microbiome changes and may carry long-term metabolic effects into adulthood.

**Conclusions:**

We present correlations between second-generation antipsychotics, gut microbiota alterations and weight gain, and suggest some mechanisms that may link them. A better understanding of the underlying mechanisms may lead to the design of improved treatments for psychotic disorders with fewer harmful side effects.

## Background

Recent microbiome research has highlighted the crucial roles of bacteria residing within our gut in affecting human metabolism, weight, inflammatory state, and overall health [1]. Distinct disease states display pronounced alterations in microbial composition and diversity, including mental disorders. Different medications have also been shown to affect the gut microbial composition, thereby potentially affecting metabolism and immunity [2].

In this review, we focus on the effects of antipsychotic medications on the microbiome of children and adolescents. While very few studies have been carried out on this population, and most of the effects and proposed mechanisms are similar to those reported in adults, we believe that this population has increased risk for long-term microbiome changes and metabolic effects up to adulthood. The use of atypical antipsychotic drugs (also known as second-generation antipsychotics, or SGAs) has increased in the pediatric population, especially within the last decade, because of their effectiveness and much lower rates of extrapyramidal side effects compared with older generation drugs [3–5]. The effects of SGAs on the gut microbiome are specifically of interest because both these factors are known to affect metabolism and weight gain [6]. Here, we present accumulating data linking the use of SGAs in children, adolescents, and adults with gut microbiota alterations and weight gain. We present an array of severity of these effects between different SGAs, and try to explain the underlying mechanisms. While some pieces of the puzzle remain missing, a picture is emerging in which SGAs have strong effects on the microbiota, leading to weight gain and potential increased risk for further morbidities including obesity, type 2 diabetes and cardiovascular disease. Understanding the mechanisms involved in SGA-induced weight gain is likely to enable improved treatments to reduce these unwanted side effects.

## Main text

### The use of atypical antipsychotic agents in children diagnosed with mental health disorders

Atypical, or second-generation antipsychotic drugs (SGAs) are commonly prescribed as a first-line treatment for psychotic disorders (e.g., schizophrenia and bipolar disorder). They are also often prescribed for children and adolescents with behavioral problems (e.g., oppositional defiant disorder and conduct disorder), borderline personality disorder, eating disorders, autism and other psychiatric conditions [7]. While some countries have approved SGAs only for certain clinical indications, off-label use is also quite common [8]. These medications are sometimes prescribed for children as young as 2 years of age [9]. However, importantly, approximately 20% of patients who are prescribed SGAs are resistant to treatment, and in many cases, SGAs have unwanted side effects, such as weight gain [10].

### Atypical antipsychotics and weight gain in children and adolescents

Although the precise etiology is poorly understood, SGAs are associated with induced weight gain, adipose tissue accumulation, and metabolic side effects. Weight gain is rather acute, with a significant increase usually seen within 12 weeks of initiation of treatment [8, 11]. However, the extent of the weight gain varies between individuals; some gain significantly more weight than others [8]. Weight and fat gain may eventually lead to metabolic syndrome and type 2 diabetes, manifested by insulin resistance, glucose intolerance, dyslipidemia and hypertension, and an increased risk for cardiovascular illness [12–14]. The risk of weight gain is higher in young children than adolescents and young adults [15, 16]. Additional predictors of SGA-induced weight gain include newly initiated treatment, high doses, long-term administration, use of medical cannabis during SGA treatment, and low initial body mass index (BMI) at the beginning of treatment [17]. Interestingly, not all patients suffer from SGA-induced weight gain to the same extent, suggesting that additional factors may determine susceptibility [8]. Studies investigating gender-dependent metabolic effects of olanzapine and other SGAs were inconclusive; most studies found the prevalence of SGA-induced weight gain to be greater in females than males, and defined low-BMI young females as an at-risk group for this adverse effect of SGAs, but some studies did not find this correlation with gender [18, 19]. Pramyothin and Khaodhiar [20] demonstrated that SGA-induced weight gain is caused by increased food consumption and the drugs’ strong influence on eating behavior, rather than decreased energy expenditure. Similarly, numerous studies have shown increased caloric intake and appetite in patients receiving SGA treatment [21]. However, additional mechanisms, including slow metabolic rates, have also been suggested [22].

It has been found in both children and adults that the SGAs olanzapine and clozapine have the most significant effect on weight gain, while risperidone and quetiapine have a lesser effect, aripiprazole is even weaker, and ziprazidone has the least effect on weight gain [11, 15, 23, 24].

### SGAs can cause metabolic side effects such as diabetes and cardiac disease

As mentioned earlier, another concerning issue while taking SGAs is the higher probability of developing type 2 diabetes, especially among the pediatric population [25]. Studies showed that SGAs lead to decreased insulin secretion and less effective glucose metabolism. Indeed, glucose levels were elevated after taking some types of SGA medications [26]. In a comparison of children aged 9–18 taking SGAs and medication-naïve children, the insulinogenic index and insulin sensitivity index-2 was much lower in quetiapine-treated children compared to the naïve group [27]. Studies in mice showed similar results, but only with clozapine [28]. While some of the metabolic effects related to SGAs may be secondary to weight gain, studies show that SGAs also have direct effects on insulin resistance and glucose dysregulation that are independent of weight gain, and even of psychiatric morbidity [29].

It has been suggested that SGAs may increase the risk for cardiac disease (e.g., cardiac arrhythmia and acute coronary syndrome) [7, 30]. In patients with schizophrenia, it has been shown that taking SGAs potentially causes an elevated risk of acute coronary syndrome, particularly at the start of therapy [31]. Additionally, in a meta-analysis of 176 reports of SGA side effects, cardiac abnormalities (e.g., cardiac arrhythmias, prolonged QT intervals and orthostatic hypotension) observed in electrocardiograms were found to be relatively common side effects [7]. An increased risk for ventricular arrhythmia has also been associated with the use of antipsychotic drugs [30]. Such clinical manifestations may be a serious risk factor for sudden cardiac death [30]. However, these findings remain controversial because many studies have found no effects of SGAs on parameters such as QTc [32], specifically in children and adolescents [33]. When testing the incidence of major cardiovascular events, some antipsychotic regimens appeared to increase incidence, although this was mostly in older adult patients [34].

### Mechanisms of atypical antipsychotic-induced weight gain

Atypical antipsychotics are known to affect body weight through different and complex mechanisms [6], including direct peripheral pathways through fatty tissue, liver and muscles, and via interactions with neurotransmitter receptors such as serotonergic, histaminergic, dopaminergic, adrenergic, cannabinoid and muscarinic receptors [35].

SGAs affect neurohormone receptor signaling: they have antagonistic effects on hypothalamic receptors such as H1R (histaminergic receptor), and on the serotonergic receptors 5-HT2a and 5-HT2c [36, 37]. Olanzapine and clozapine influence metabolic regulation [6] and have the most powerful antagonistic effect on the hypothalamic H1Rs, correlated with both short-term and long-term SGA-induced obesity. Initial effects of blocking hypothalamic H1Rs include activation of AMP-activated protein kinase (AMPK), a well-known feeding regulator, thereby increasing caloric intake. Long-term effects may reduce thermogenesis in brown adipose tissue and decrease lipolysis, while increasing lipogenesis in white adipose tissue [38]. Treatment with betahistidine (an H1R agonist and H3R antagonist) prevented olanzapine-induced weight gain in mice [39]. Antagonist effects on the serotonergic receptor may also influence metabolism, since 5-HT2c is the primary receptor influencing appetite, thereby also affecting control of energy balance [40]. Ballon et al. proposed that antipsychotics might have partial agonistic affinity to pancreatic beta-cell receptors (5-HT2a and 5-HT2c receptors, dopamine D2-like receptors [D2Rs], and muscarinic M3 receptors), thus causing dysfunction of these cells, leading to diabetes [36].

#### Peptide hormones may mediate SGA-induced weight gain

Antipsychotics were shown to modulate ghrelin and leptin hormonal signaling, cause glucose and lipid dysregulation, alter tumor necrosis factor-alpha (TNFα) cytokine production, and affect adiponectin [41]. In one animal experiment, rats receiving olanzapine for 3 weeks showed increased visceral fat, decreased activity, and an increase in macrophage infiltration to adipocytes compared with the control group. Interestingly, female but not male rats receiving olanzapine showed significant weight gain, as well as decreased ghrelin levels; this might result from negative feedback attributed to increased food and water intake. Surprisingly, greater weight gain was discovered in females receiving lower rather than higher doses of olanzapine [42]. Several human studies have also found correlations between atypical antipsychotics, expression of peptide hormones, and induced weight gain [43–45]. One such study of 117 children and adolescents found increased insulin levels, insulin resistance (HOMA-IR), and leptin levels following 3 and 6 months of SGA treatment [45]. In a meta-analysis including 1156 participants, SGAs significantly elevated leptin levels [43]. Another study in men with schizophrenia found decreased serum adiponectin levels and insulin resistance in patients versus healthy controls [44]. A third study of 112 adult patients receiving SGAs correlated treatment-associated weight-gain and elevated serum ghrelin, rather than leptin deficit, compared with healthy controls [46]. A recent study comparing metabolic parameters between antipsychotic-induced weight gain and non-pharmacological obesity in youth found that leptin and ghrelin were related to BMI rather than to antipsychotic exposure. On the other hand, antipsychotic-induced weight gain was associated with higher C-peptide, glucose-dependent insulinotropic polypeptide, and adipsin [47].

#### SGAs may cause mitochondrial dysfunction

SGAs have been found to inhibit mitochondrial energy generation by downregulating genes encoding subunits of the electron transport chain (ETC) complexes, decreasing related enzyme activity and ATP levels, and significantly decreasing oxygen consumption in cells [48]. These effects on mitochondrial function may increase the risk of metabolic syndrome associated with SGAs.

#### Genetic determinants of SGA-induced weight gain

Several studies indicate that hereditary factors may be involved in SGA-induced weight gain [9]. The level of olanzapine-induced weight gain in mice was found to be strain-dependent when tested in eight different mouse strains: one strain (C57BL/6 J) was found to be specifically susceptible to olanzapine-induced weight gain [49]. This outcome correlates with additional results suggesting genetic influences on SGA-induced weight gain in humans. In a study of monozygotic twins and siblings receiving SGAs, the influence of hereditary factors on weight gain was between 60 and 80% [50], supporting the notion that genetic factors may affect susceptibility to metabolic side effects in SGA-treated children. The mechanisms underlying this susceptibility are not quite clear, but might be related to serotonin receptors and additional hormones [15]. In support of this, new studies have found genetic risk factors for SGA-induced weight gain, including polymorphism near the genes for melanocortin-4-receptor (MC4R), serotonin-2-receptor (HTR2C), and the leptin gene linked to fat mass. Despite these results, there remains no practical way to identify genetically prone patients to develop treatments with minimal metabolic side effects [51].

Recently, with the emergence of microbiome research focusing on the microbial populations within the human body, it was proposed that SGA-induced weight gain is mediated by the microorganism composition of the gut. Numerous findings have suggested the influence of the microbiota on SGA-induced weight gain through the connection between microbiome composition, obesity, and disease states.

### The gut–brain axis connects the gut microbiome and psychiatric disorders

The gut–brain axis implies bidirectional communication and connection between the gut and the brain. The influence of the brain on the gut may seem more obvious, as it is well known that stress and many mental disorders (especially neuroses, stress-related disorders, somatization disorders, autonomic dysfunction, and anxiety) include gastrointestinal symptoms [52]. However, it is evident that the gut microbiota also has a great influence on brain development and functionalities. Thus, gut microbiota may affect appetite, emotions, and cognition, and contribute to or prevent various mental conditions, including major depression disorder (MDD), schizophrenia, and autism [53–55]. Indeed, the gut microbiome is altered in various psychiatric disorders. Several studies have reported deviation from a healthy microbiome composition in treatment-naïve patients diagnosed with schizophrenia, bipolar disorder, and MDD with psychotic features compared with healthy individuals [56, 57]. However, the specific alterations were inconclusive. Comparing the microbial composition of 115 patients with bipolar disorder to 64 healthy individuals showed a significantly lower representation of bacteria from the *Faecalibacterium* genus in bipolar patients, associated with poorer health [58]. This genus was also reportedly decreased in patients with MDD [59], along with reduced microbial diversity, increased *Bacteroidetes* bacteria, and decreased *Firmicutes* bacteria. Beyond the alterations in gut microbiota compositions seen in MDD patients, a causal relationship was found when germ-free (GF) mice were transplanted with microbiome from MDD patients and showed depression symptoms [60]. Thus, gut microbiota could be a direct cause of MDD [61]. Very few studies have been conducted on the microbiome in children and adolescents suffering from psychiatric disorders. Studies on children with autism spectrum disorder (ASD) have shown distinct differences in fecal microbiota composition compared with healthy controls [62, 63]. This is especially intriguing because many children with ASD also suffer from gastrointestinal side effects. Additional studies have shown distinct differences between the microbial composition in children with ADHD versus controls [64], and in cases of eating disorders [65, 66].

The principal pathways of the gut–brain axis include action through the vagus nerve, involving the endocrine system, the hypothalamic–pituitary–adrenal (HPA) axis, neurotransmitter pathways, metabolites, and immune system components [67]. Recently, gut bacteria were shown both to produce and respond to neurohormones such as serotonin, dopamine and norepinephrine [68]. In fact, 90% of the body’s serotonin is found in the gut. Serotonin levels may further affect microbial composition. In a study of mice lacking a serotonin transporter, causing elevated serotonin levels in the gut, there were distinct microbiota compositional alterations, including formation of populations resembling those of depressed patients [69]. Serotonin can also promote growth and virulence in certain bacteria [70, 71]. Dopamine is another neurohormone produced by bacteria including *Bacillus* and *Serratia*, although little is known about its function in these microorganisms [68]. Free dopamine levels appear significantly lower in GF mice than in conventional mice, reinforcing a connection between dopamine and microbiota [72]. As dietary dopamine is linked to glucose homeostasis [73], it would be intriguing to study the interconnections between diet, gut microbiota, dopamine, and glucose homeostasis. Additional evidence links gut microbiota with modulation of the stress hormones corticosterone and adrenocorticotropic hormone (ACTH) in response to mild stress [55].

Therefore, one hypothesis to explain SGA-induced weight gain suggests that the antagonistic effects of SGAs on neurohormone receptors (5-HT2C, muscarinic, and H1) alter the gut microbiota composition, which in turn leads to increased weight gain. Alternatively, the change in microbiota composition caused by SGAs may be a secondary effect resulting from increased caloric intake. Changes in dietary intake have previously been reported to derive a change in microbial composition [74–76]. Nonetheless, the full connection between neurohormones, SGAs, and microbiota remains to be elucidated.

An additional pathway that may link the brain and the gut in psychiatric disorders is through the immune system. Many medical conditions, such as depression, include chronic low-grade inflammation, which affects microbial composition and gut permeability [61, 77]. More specifically, several changes in cytokine levels (specifically, increases in pro-inflammatory and decreases in anti-inflammatory cytokines) are associated with depression. Inflammation increases depression morbidities by mechanisms involving inhibition of the negative feedback of the HPA axis, increased permeability of the blood–brain barrier, reduced synthesis of 5-HT, and disturbance of glutamatergic pathways [63]. However, inflammation also affects the gut microbiota composition, and may lead to changes in gut permeability. In turn, increased gut permeability may lead to release of lipopolysaccharide, which can further induce pro-inflammatory cytokines (e.g. IL-6, IL-1) and norepinephrine levels [78]. The interconnections between the gut microbiota and immune system have been extensively studied, and clearly, the gut microbiota at young ages is crucial for educating the immune system, brain development, and brain function [79]. Therefore, any dysbiosis during infancy or early childhood may have life-long implications on immune function. Pathways of the gut–brain axis, specifically at the stages of adolescence, have not been extensively studied. However, a study highlighting functional differences between the microbial composition of children aged 7–12 and adults, found lower expression of microbial genes linked to inflammation in children, including genes involved in lipopolysaccharide biosynthesis [80].

### Gut microbiome and obesity

The association between gut microbiota and obesity has been primarily investigated in rodent models by creating GF mice and the availability of fecal microbiota transplants (FMT). When fecal samples were transplanted from conventional mice into GF mice, the adiposity of these mice rose significantly, even after reducing the number of calories consumed. These experiments support an active role of the gut microbiota in obesity in mice [81]. Similar results were found in a study of human twins discordant for obesity [82]; microbiota composition differs between obese and lean individuals and supports the possibility of microbiota-induced obesity.

In obesity, the gut microbiota is generally less diverse, associated with adiposity and dyslipidemia, impaired glucose homeostasis and low-grade inflammation [83]. A high relative ratio of *Firmicutes* to *Bacteroidetes* bacteria has been associated with obesity in many, but not all, rodent and human studies [84–86]. This ratio was reported to decrease when overweight people start either a low fat or low carbohydrate diet [87]. The relative abundance of *Actinobacteria* also appears higher in obese people [88]. While there are differences in the precise bacterial compositions between obese individuals, their microbial gene expression and related metabolic functions may be more common [88, 89].

A variety of mechanisms link gut microbiota composition with obesity. One such pathway is altering the amount of energy harvested from dietary intake by fermenting dietary fibers into short chain fatty acids (SCFA), inducing lipogenesis, influencing satiety, and decreasing energy expenditure [90–92]. Additionally, various microbiota species are thought to affect hormone signaling pathways, including those of leptin and ghrelin [55], and some are even presumed to play a role in modulating host epigenetics [93]. Since the gut microbiota can trigger the host’s innate immune system, certain compositions may also promote production of pro-inflammatory signals associated with obesity, insulin resistance and other metabolic dysfunctions [94]. While these functions of the microbiota provide clues to the precise roles of the microbiota in obesity, the exact pathways remain to be determined.

### Linking the gut microbiome and antipsychotic drug-induced obesity

Several studies have explored the connection between antipsychotic drug-induced obesity and gut microbiota (see Tables 1 and 2). Most studies concentrated on metabolic and microbiota compositional outcomes of risperidone and olanzapine treatments, the two most widely used SGAs in patients of all ages that lead to significant induction of weight gain, as described above. Several studies have been performed in rats and mice to allow for controlled lab conditions including diet, which can lower diversity between subjects and highlight the specific effects of SGAs. Most studies in both humans and mice have shown changes in the gut microbial communities following SGA treatment. An increase in the *Firmicutes* to *Bacteroidetes* ratio following use of olanzapine or risperidone has been consistently reported in several studies [22, 42, 49, 95, 96]. However, inconsistent results were found in studies investigating the effects of risperidone or olanzapine on the relative abundance of bacteria from the *Actinobacteria* phylum. Some studies showed a rise in *Actinobacteria* abundance, correlated with increased weight gain and visceral fat [22], agreeing with previous findings correlating *Actinobacteria* with obesity [88]. In contrast, another study found a decrease in *Actinobacteria* abundance in olanzapine-treated mice [42]. A deeper look at the level of classes and species taxonomy revealed that these changes correlate with gut bacterial functions previously suggested to be obesogenic. For example, in mice, risperidone treatment increased the relative abundance of the *Erysipelotrichaceae* family, previously reported to have high abundance in mice fed a high-fat diet [98]. Additionally, risperidone treatment increased levels of the *Mollicutes* class, including species rich in metabolic genes for glycan and simple sugar fermentation [99], while decreasing relative abundance of bacterial species previously reported to correlate with ‘lean gut microbiota’, such as *Alistipes* spp. and *Akkermansia* spp. [82, 100]. These bacterial changes in risperidone-treated mice correlated with significant weight gain, with no increased food intake, and are thereby assumed to result from suppressed energy expenditure [22].

**Table 1:** Studies of second-generation antipsychotics and microbiota in rodents

Cohort description	Drug(s)	Microbial taxonomy alterations	Microbial diversity alterations	Host metabolic alteration	Country	Reference
Rats (treated in high/low doses vs. control)	Olanzapine	*Firmicutes* ↑, *Bacteroidetes*↓, *Proteobacteria*↓, *Actinobacteria*↓ (in female rats only)	Diversity ↓	In females: Weight gain in females, rise in food intake, increased liver size, increased visceral fat. In males and females: Increased visceral fat, increased macrophage infiltration to adipose tissue, decreased locomotion	Ireland	[42]
Rats (treated vs. control)	Olanzapine	*Firmicutes* ↑, *Bacteroidetes* ↓, *Proteobacteria*↓		Rapid weight gain, increased visceral fat, increased macrophage infiltration to adipose tissue, increased free fatty acids in plasma.	Ireland	[95]
Rats (treated vs. treated with antibiotics)	Olanzapine and antibiotics	Following antibiotics: *Firmicutes* ↓, *Bacteroidetes* ↑, *Proteobacteria*↑		Following antibiotics: less weight gain	Ireland	[95]
Mice (treated vs. control from 8 strains)	Olanzapine and high fat diet	*Erysipelotrichi* ↑, *Gammaproteobacteria* ↑, *Bacteroidia* ↓		Weight gain differing by strain	USA	[49]
Mice (treated germ-free)	Olanzapine (with/without fecal transplant)			Weight gain only after fecal transplants from conventionally raised mice	USA	[49]
Female mice (treated vs. control)	Risperidone	*Erysipelotrichaceae* family ↑, *Mollicutes* class ↑, *Alistipes* spp. ↑, *Actinobacteria* phylum ↑		Weight gain, reduction in energy expenditure	USA	[96]

**Table 2:** Studies of second-generation antipsychotics and microbiota in humans

Cohort description	Mean age (years)	% Males	Drug(s)	Microbial taxonomy alterations	Microbial diversity alterations	Host metabolic alteration	Country	References
Adolescents (5 commencing treatment vs. 10 healthy controls)	11.7	100	Risperidone	*Bacteroidetes*: *Firmicutes* ratio↓	Diversity ↑	Higher BMI	USA	[96]
Adolescents (18 treated over a year vs. 10 healthy controls)	12.2	100	Risperidone	*Bacteroidetes*: *Firmicutes* ratio↓, *Proteobacteria* ↑, *Actinobacteria* ↑, *Verrucomicrobia* ↓		Higher BMI	USA	[96]
Adults (117 bipolar disorder patients: 49 treated with SGA, 68 non-treated)	46	26	Clozapine, olanzapine, risperidone, quetiapine, asenapine, ziprasidone, lurasidone, aripiprazole, paliperidone, iloperidone	*Akkermansia*↓ in non-obese treated vs. untreated patients	Diversity↓	Higher BMI	USA	[97]
Age not defined (41 patients with a first episode of schizophrenia and normal body weight)	23.1	56%	Risperidone	*Bifidobacterium* spp. ↑		Higher BMI, weight gain	China	[56]

A study on drug-naïve, normal-weight, first-episode young schizophrenia patients revealed significant alterations in abundance of microbiota species following 24 weeks of risperidone treatment [56]. A deviation to the ‘obese gut microbiota’ was also reported in a longitudinal study monitoring metabolic and microbiota changes throughout 10 months of risperidone treatment in five male psychiatric disorder adolescents aged 9–13 [96]. Specifically, treated patients presented a gradual increase in *Firmicutes* to *Bacteroidetes* ratio, correlating with a rise in BMI. Additionally, the gut microbiome of participants treated with risperidone for over a year was enriched with genes for SCFA and serotonin metabolism [96].

Lower species diversity, another characteristic of the ‘obese gut microbiota’ [88], was found in 49 bipolar disorder patients treated with various SGA drugs versus 68 untreated patients. Interestingly, female participants receiving treatment showed a more significant reduction in diversity than male participants; a result that correlates with earlier findings of gender-dependent SGA-induced weight gain [97].

Olanzapine may have a direct antimicrobial effect on some microbiota species, as demonstrated in an experiment monitoring the growth of two common gut bacterial habitants (*Escherichia coli* and *Enterococcus faecalis*). In this in vitro experiment using various doses of olanzapine, *E. coli* exhibited complete growth inhibition in an environment with very high levels of olanzapine (580 μm/ml), while *E. faecalis* showed a growth delay [49]. Negative selection of some bacterial species, but not others, may greatly influence the gut microbiota composition and reduce diversity.

One way to test the roles of the microbiota is to reduce its activity using antibiotics. In a study of female rats, olanzapine treatment led to significant metabolic changes, including weight gain, increase in food and water intake, elevation of plasma free fatty acids, and the liver lipogenic enzyme fatty acid synthase. However, when broad-spectrum antibiotics were administered with olanzapine treatment, significant decreases were observed in all of these metabolic changes. There were also distinct differences in gut microbiota compositions between olanzapine and antibiotic co-treated rats versus rats treated with olanzapine alone. For example, the relative abundance of bacteria from the *Firmicutes* phylum increased following olanzapine, but not when co-administered with antibiotics. Additionally, the relative abundances of *Bacteroidetes* and *Proteobacteria* decreased following olanzapine, but not when co-administered with antibiotics [95].

The role of the gut microbiota in olanzapine-induced weight gain was further demonstrated by testing GF versus conventional mice consuming a high fat diet. While the conventional mice treated with olanzapine showed significant weight gain compared with placebo-treated mice, the olanzapine-treated GF mice showed no weight gain compared with placebo. Furthermore, when GF mice were transplanted with microbiota, a significant difference in weight gain was demonstrated between mice transplanted with olanzapine-treated versus control gut microbiota [49]. Taken together, these results show that the microbiota forms a fundamental link between SGA treatment and weight, and suggest an active role of the gut microbiota in inducing weight gain.

### Special considerations regarding SGA use in adolescents

While the connection between SGAs, weight gain and changes in gut microbiota is intriguing for populations of all ages, the child and adolescent patient groups require more careful attention. First, the microbiota composition of children and adolescents differs from that of adults. The common belief is that, by 3 years of age, our gut microbiota stabilizes and becomes adult-like; however, newer research shows that maturation of the gut microbiota is a more extended process, with phylogenetic differences decreasing with age [101], and that distinct differences remain between the adolescent and adult gut microbiota [80]. The gut microbiota of children and adolescents is less stable and less diverse than the adult microbiota [102]. Thus, it may be viewed as a transition state between infant and adult microbiota in both its bacterial structure and functionality. Therefore, the influence of external factors on gut microbiota of children and adolescents may be more extreme than in adults, and have long-term effects, shaping the adult bacterial compositions and influencing the tendency to gain weight. Additionally, as the interconnections between hormones and the gut microbiota unravel, adolescence may be a period in which sex hormones affect the microbiome composition. Both male and female hormones have been associated with microbial components; for example, the intestinal microbiota composition changes in response to manipulation of estrogen receptor (ER-β) [103], and in response to hormonal imbalance, such as in polycystic ovary syndrome (PCOS), or pregnancy [104, 105]. Testosterone levels have been shown to rise in the presence of microbes, in a non-obese diabetic (NOD) mouse model [106].

Next, adolescence is a critical period for brain development, including both neurobiological and social-affective development. Since there is proof that the microbiome affects brain structure and function in early life, and only a few studies have tested these aspects at later developmental stages, it would be promising to test microbiota effects on brain function in adolescence [107]. Finally, it is known that obese children have a major risk of becoming obese adults, suggesting that weight and metabolic state at a young age have long-term effects influencing later life [108]. These may be mediated by microbiota.

Another aspect to be considered specifically in pediatric and adolescent populations is the negative social impact of weight gain itself. It has been shown that SGA-related weight gain is even more dramatic in children and adolescents than in adults [109]. Children and adolescents taking SGAs may already suffer from low self-esteem and the stigma of being different from their peers. Increased weight gain may enhance this perception. Therefore, SGA-induced weight gain may increase the risk of major psychological and physiological outcomes during childhood and adolescence [110, 111]. This may also have effects on compliance to medication, requiring alternative treatments that do not cause weight gain as a side effect.

### Current directions for alleviating SGA-induced weight gain

To date, despite the base of evidence linking weight gain to SGA treatments, there is no standard treatment for the associated weight gain and metabolic side effects. If severe metabolic side effects occur (e.g. type 2 diabetes or metabolic syndrome), they are treated individually, as secondary comorbidities. However, while they require additional testing, several treatment directions have been suggested. Among these are glucagon-like peptide 1 receptor agonists (GLP-1RAs) and metformin. GLP-1RAs are routinely used to treat type 2 diabetes, and are associated with significant weight loss [112]. Since GLP-1 levels have been found to rise following SGA treatment [113], and are associated with insulin resistance and hyperglycemia, treatment with GLP-1RA may reduce these metabolic side effects, as seen in rodent [114] and human studies. In a meta-analysis including 164 patients in three clinical studies, which tested GLP-1 receptor agonists for the treatment of antipsychotic-associated weight gain, this treatment was shown to be safe and efficient, although larger trials are recommended [115]. Two small pediatric clinical trials have suggested that GLP-1RA treatment may also be useful in the adolescent population [116]. Metformin is another drug commonly used for treatment of type 2 diabetes, with proven effects in reducing glucose levels, improving insulin resistance, lowering total cholesterol levels, and reducing weight gain in schizophrenia patients [117]. The use of metformin to alleviate SGA side effects has been extensively studied, and, in a meta-analysis including 40 studies [118], was shown to be effective. It was also suggested that metformin may be effective for combating weight gain associated with SGAs in children and adolescents [119].

Other drugs that have been effective in controlling SGA-induced weight gain include topiramate, sibutramine, aripiprazole and reboxetine [118, 120]. Aripiprazole and sibutramine act by reducing lipid levels, as does metformin. Aripiprazole is also an antipsychotic, with partial effects on dopamine and serotonin receptors. Alternatively, reboxantine is a norepinephrine reuptake inhibitor, which has been reported to reduce appetite and lower olanzapine-related weight gain [120], and topiramate is an anti-epileptic drug that reduces appetite, inhibits lipogenesis, and was shown to reduce SGA-induced weight gain in several adult clinical studies [121–123]. Although not yet broadly tested as co-treatment together with SGAs in children and adolescents, it has been tested in a clinical study in adolescents with juvenile bipolar disorder for replacing standard medication [124].

While several methods to alter the gut microbiome (including consumption of probiotics, prebiotics, and fecal transplants) have been suggested for therapeutic purposes in a large variety of disease states, their precise beneficial components and effectiveness remains to be carefully tested. A study testing the prebiotic B-GOS in rats receiving olanzapine suggested benefits in preventing weight gain and on cognitive function, perhaps via alteration of cytokine levels (e.g. TNFα) and levels of circulating acetate [125]. Some studies of schizophrenia patients receiving antipsychotics have shown that probiotics may alter immune parameters [126], and reduce gastrointestinal symptoms [127]; however, weight gain was not tested in these studies. Ongoing clinical trials are testing the efficacy of probiotics on alleviating SGA-induced weight gain. A better understanding of the precise roles of microbes in SGA-related weight gain is required to develop such specific microbial treatments.

To date, the most effective weight reduction interventions in schizophrenia patients appear to be lifestyle counseling and exercise, as presented in a recent meta-review and meta-analyses of clinical trials [117].

## Conclusions

**Fig. 1:**
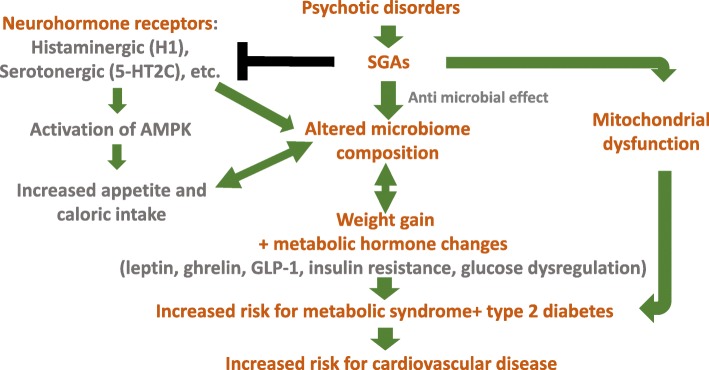
Influences of antipsychotic medication on microbiome composition, weight gain, and associated morbidities. (SGAs, second-generation antipsychotics; GLP-1, glucagon-like peptide 1; AMPK, AMP-activated protein kinase)

Much progress has been made in characterizing SGA-induced weight gain and the involvement of microbiota in this process. It appears clear that SGAs lead to significant weight gain in most patients, including adults, adolescents, and children. The role of the microbiome in this process has been demonstrated in numerous experiments, both in humans and in rodent models, and several mechanisms have been proposed to explain this connection (Fig. 1). A better understanding of these specific pathways and related risk factors is essential, given the growing clinical use of SGAs within both adult and pediatric populations.

However, many open questions remain. Are there initial microbiome compositions that are more sensitive to SGA treatment and therefore predict enhanced weight-gain? Deciphering such microbial biomarkers may help to tailor the best treatments for patients, while minimizing weight and metabolic side effects. Why do different SGA compounds lead to varying levels of weight gain? What are the precise mechanisms of action, and can these side effects be eliminated? Answers to all these questions may lead to meaningful medical outcomes, including improved treatments, more personalized medicine, prediction of side effects, and perhaps ways to overcome them.

Another aspect not yet studied in depth is whether certain microbes can influence the therapeutic efficacy of SGA treatment. Although SGA treatments are commonly prescribed, relatively high risks of relapse during continuous treatment remain, with efficacy rates that are far from ideal. However, there is no accurate method to predict which patients will respond best to treatment. It is possible that different microbes or microbial compositions may play antipsychotic roles, thereby improving the efficacy of SGA treatment.

Additionally, the specific characteristics of the child and adolescent microbiotas have not been carefully considered to test for unique effects that may be different from those in adults. We strongly believe that more research is needed to better treat this important patient population and enable fewer long-term metabolic effects.

Children and adolescents suffering from various mental health disorders are often treated with SGAs. While these drugs decrease psychotic symptoms, they usually lead to increased weight gain. Multiple studies have indicated that this SGA-induced weight gain occurs via alteration of the microbiome composition. However, it is not yet clear how SGAs affect the microbiome. One suggested mechanism involves blockage of histaminergic (e.g. H1) and serotonergic (e.g. 5-HT2C) receptors, thereby manipulating the satiety regulator AMP-K, further leading to increased appetite and caloric intake, and finally altering microbiome composition and lowering diversity. Additional mechanisms may exist, such as SGAs directly inhibiting the growth of specific microbial species, and direct effects on mitochondrial dysfunction. Whether directly or indirectly, SGAs also lead to metabolic hormone imbalance, altering levels of leptin, ghrelin, GLP-1, insulin, and glucose.

Weight gain and altered microbial composition may lead to additional morbidities including development of metabolic syndrome, type 2 diabetes, cardiovascular disease, and – potentially – even worsening of the psychological state and mental health disorders caused by these issues.

## Abbreviations

5-HT: 5- hydroxytryptamine
ACTH: Adrenocorticotropic hormone
AMPK: AMP-activated protein kinase
ATP: Adenosine triphosphate
BMI: Body mass index
D2R: Dopamine D2-like receptor
ER-β: Estrogen receptor
ETC: Electron transport chain complex
FMT: Fecal microbiota transplants
GF: Germ-free
GLP-1: Glucagon-like peptide 1
H1R: Histaminergic-1 receptor
HPA: Hypothalamic–pituitary–adrenal
HTR2C: Hydroxytryptamine − 2-receptor
MC4R: Melanocortin-4-receptor
MDD: Major depression disorder
NOD: Non-obese diabetic
PCOS: Polycystic ovary syndrome
SCFA: Short chain fatty acid
SGA: Second-generation antipsychotic
TNFα: Tumor necrosis factor alpha
